# Artificial Intelligence in Sports Medicine: A Decision-Centered Framework for the Future Sports Physician

**DOI:** 10.3390/diagnostics16101448

**Published:** 2026-05-09

**Authors:** Stefano Palermi, Rita Pucciatti, Nor-Eddine Regnard, Ali Guermazi, Fabiano Araujo, Andrea Demeco, Yosra Mekki, Giuseppe D’Antona, Alessia Guarnera, Simone Cerciello, Matteo Guzzini, Marco Vecchiato

**Affiliations:** 1 Departmental Faculty of Medicine and Surgery, UniCamillus-Saint Camillus International University of Health Sciences, 00187 Rome, Italy; rita.pucciatti@gmail.com (R.P.); guarneraalessia@gmail.com (A.G.); simone.cerciello@unicamillus.org (S.C.); matteo.guzzini@unicamillus.org (M.G.); 2 Gleamer, 75010 Paris, France; noreddine.regnard@gleamer.ai; 3 Réseau d’Imagerie Sud Francilien, Clinique du Mousseau Ramsay Santé, 91042 Evry, France; 4 Department of Radiology, VA Boston Healthcare System, Boston University School of Medicine, Boston, MA 02130, USA; guermazi@bu.edu; 5 FCA Consulting Services, Belo Horizonte 30180-106, MG, Brazil; araujo@fcadata.com; 6 Physical and Rehabilitative Medicine, Department of Medical and Surgical Sciences, University of Catanzaro “Magna Graecia”, 88100 Catanzaro, Italy; andreademeco@hotmail.it; 7 College of Medicine, Qatar University, QU Health, Doha P.O. Box 2713, Qatar; yusramagdi@gmail.com; 8 Department of Public Health, Experimental and Forensic Medicine, University of Pavia, 27100 Pavia, Italy; giuseppe.dantona@unipv.it; 9 Sports and Exercise Medicine Division, Department of Medicine, University of Padova, 35128 Padova, Italy; marcovecchiato.md@gmail.com; 10 Department of Theoretical and Applied Sciences, eCampus University, Novedrate, 22060 Como, Italy

**Keywords:** digital health, injury prevention, musculoskeletal imaging, artificial intelligence, sports medicine

## Abstract

**Background:** Artificial intelligence (AI) is rapidly transforming healthcare, with increasing applications in sports medicine. Advances in machine learning, deep learning, and computer vision enable the analysis of large, heterogeneous datasets derived from imaging, wearable sensors, performance-monitoring systems, and electronic health records. While these technologies offer opportunities to enhance injury prevention, diagnostic accuracy, rehabilitation monitoring, and clinical decision-making, their integration into athlete care remains complex and context-dependent. **Methods:** A structured narrative review of the PubMed/MEDLINE database was conducted to identify clinically relevant AI applications in sports medicine. The search focused on key domains including injury risk prediction, musculoskeletal imaging, rehabilitation monitoring, return-to-play assessment, performance management, and clinical workflow support. Evidence from original studies, reviews, methodological reports, and regulatory documents was qualitatively synthesized to provide an overview of current applications, methodological limitations, and decision-level implications. **Results:** AI demonstrates growing utility across multiple domains of sports medicine. Machine learning models can identify complex, non-linear relationships among training load, physiological responses, and injury risk, though their predictive performance varies widely and is often limited by dataset heterogeneity and a lack of external validation. In musculoskeletal imaging, AI-based algorithms support automated detection and quantification of abnormalities, with performance in selected tasks approaching that of expert readers, yet remaining task-specific and context-dependent. Emerging applications include movement analysis and rehabilitation monitoring through wearable sensors and computer vision systems, as well as data-driven support for return-to-play decisions and clinical workflow optimization. However, current evidence highlights important limitations, including algorithmic bias, limited generalizability, poor interpretability, and the risk of misapplication in complex clinical decision-making contexts. **Conclusions:** AI is likely to become an important decision-support layer in sports medicine by enabling data integration and longitudinal monitoring. However, model performance does not necessarily translate into improved clinical outcomes, and AI-generated predictions remain probabilistic and context-sensitive. Consequently, clinical decisions—particularly high-stakes processes such as return-to-play—require structured integration of AI outputs within a broader clinical framework. The sports physician remains central as a human-in-the-loop integrator, responsible for contextualizing AI-derived information, mitigating potential errors, and ensuring safe, individualized athlete management.

## 1. Introduction

Artificial intelligence (AI) is progressively reshaping modern healthcare by expanding the capabilities of diagnostics, monitoring, risk stratification, treatment personalization, and patient engagement [1,2]. Beyond improving efficiency or automating routine tasks, AI is increasingly influencing the cognitive processes underlying medical decision-making, altering how clinicians interpret complex data, manage uncertainty, and integrate evidence into practice [3]. Sports medicine represents a particularly demanding setting for the implementation of AI-driven technologies [4]. Clinical decisions in this field are frequently time-sensitive and require the integration of heterogeneous and continuously evolving information, including physiological signals from wearable devices, biomechanical and motion-capture assessments, musculoskeletal imaging, and longitudinal clinical records [5]. Machine learning (ML) and deep learning (DL) approaches are well-suited to address this complexity, as they can model non-linear relationships across multimodal datasets and support probabilistic risk estimation beyond traditional human pattern recognition [6,7]. Nevertheless, these outputs remain inherently probabilistic and may be misinterpreted or over-relied upon if not adequately contextualized within the clinical scenario.

Traditionally, sports physicians have relied on clinical examination, contextual knowledge of the athlete, and experiential judgment to guide diagnostic and therapeutic decisions [8]. With the growing integration of AI-based systems into injury risk assessment, imaging interpretation, rehabilitation monitoring, and performance management, this role is progressively evolving. Rather than replacing clinical expertise, AI functions as an additional analytical layer that supports the integration and longitudinal interpretation of complex athlete data [1]. This shift introduces new challenges, including the risk of inappropriate decision-making driven by algorithmic outputs, limited generalizability across athletic populations, and the need to balance data-driven insights with clinical reasoning and athlete-specific contextual factors [9].

Despite the rapid expansion of AI applications in sports medicine, the current literature remains largely fragmented, predominantly focused on technical performance metrics and single-domain applications, and gives limited attention to how AI outputs are integrated into real-world clinical decision-making. In particular, there is a lack of structured frameworks for how probabilistic model outputs should inform high-stakes decisions, such as return-to-play (RTP), where uncertainty, risk tolerance, and contextual factors play central roles.

Against this background, this narrative review examines the role of AI across non-cardiovascular domains of sports medicine, focusing on injury prevention, musculoskeletal diagnostics, rehabilitation monitoring, RTP decision-making, performance optimization, and clinical workflow support. The aim is not to provide a purely technical overview of AI technologies, but rather to critically explore how AI is reshaping clinical reasoning, to identify current limitations and potential sources of error, and to propose a decision-centered perspective on the integration of AI into sports medicine practice. Particular emphasis is placed on the evolving role of the sports physician as a human-in-the-loop integrator, responsible for contextualizing AI-derived outputs within the broader clinical, ethical, and sport-specific framework of athlete care.

## 2. Methods

### 2.1. Study Design

This article adopts a structured narrative review approach to synthesize current applications of AI across clinically relevant domains of sports medicine. A narrative methodology was selected due to substantial heterogeneity across the available literature in study design, populations, data sources, AI methodologies, and outcome definitions. Under these conditions, quantitative synthesis or formal evidence grading would have been methodologically inappropriate and potentially misleading. Rather than providing a systematic comparison of algorithms or performance metrics, this review aimed to analyze how AI technologies influence clinical decision-making in sports medicine, with particular attention to the evolving roles and responsibilities of sports physicians.

### 2.2. Identification of the Research Question

The review was guided by the following overarching research question: “How is artificial intelligence influencing clinical decision-making and professional responsibilities in contemporary sports medicine practice?”

To address this question, the literature was explored across key domains of sports medicine in which AI-based tools are currently proposed or implemented, including:

- Injury risk assessment and prevention;
- Musculoskeletal diagnostic support;
- Rehabilitation monitoring;
- Return-to-play (RTP) decision-making;
- Performance and workload management;
- Clinical workflow and cognitive support.

Accordingly, the review adopts a decision-centered framework, focusing on how AI-derived outputs may inform specific clinical decisions encountered in routine sports medicine practice, rather than solely evaluating model performance.

### 2.3. Eligibility Criteria

Publications were considered eligible if they examined the application of AI-based methods—including ML and DL, natural language processing, or computer vision—within domains relevant to sports medicine practice.

Studies were included when they addressed one or more of the following areas:

- Injury risk prediction or prevention in athletes;
- AI-assisted musculoskeletal imaging or diagnostics;
- AI-based rehabilitation monitoring or movement analysis;
- AI-supported RTP assessment;
- Performance or workload monitoring systems;
- AI applications supporting clinical workflow or decision support.

Studies focusing exclusively on non-athletic general populations, animal models, or purely technical algorithm development without clear clinical applicability were excluded. Given the heterogeneity of methodologies, validation strategies, and outcome measures across the included literature, formal risk-of-bias assessment and meta-analytic procedures were not performed. Instead, the quality and limitations of the evidence base were considered qualitatively during synthesis, with particular attention to generalizability, validation strategies, and potential sources of bias.

### 2.4. Information Sources and Search Strategy

A structured literature search was conducted in the PubMed/MEDLINE database to identify relevant publications addressing AI applications in sports medicine. The search covered the period from January 2015 to December 2024, reflecting the rapid evolution of AI methodologies over the past decade.

Search terms included combinations of the following keywords:

- “artificial intelligence”;
- “machine learning”;
- “deep learning”;
- “sports medicine”;
- “injury prediction”;
- “rehabilitation”;
- “return to play”;
- “wearables”;
- “digital health”.

The search strategy was complemented by manual screening of the reference lists of relevant review articles and key publications to identify additional studies. In addition to original research articles, the review also considered systematic reviews, narrative reviews, methodological studies, consensus statements, and regulatory reports, reflecting the field’s multidisciplinary and rapidly evolving nature.

### 2.5. Study Selection and Data Synthesis

The initial search yielded approximately 1284 records, of which 1012 were screened after duplicates were removed. Following title and abstract screening, 142 full-text articles were assessed for eligibility, and 74 studies were ultimately included in the qualitative synthesis. Given the narrative nature of this review and the substantial heterogeneity across study designs, populations, and AI methodologies, a formal risk-of-bias assessment was not performed. To enhance methodological transparency, an interpretative level-of-evidence classification was assigned to studies included in Table 1 and Table 2, based on study design, validation strategy, and clinical applicability. This classification is intended to provide a pragmatic overview rather than a formal methodological appraisal.

## 3. Transformation Areas in Sports Medicine

AI is increasingly influencing sports medicine not merely through the introduction of new technologies, but by reshaping how clinical decisions are generated, supported, and contextualized [6]. Traditional approaches have largely relied on episodic assessments, population-derived thresholds, and retrospective interpretation of injury and performance data. In contrast, AI enables the integration of continuous, multimodal data streams, supporting a transition toward individualized and dynamic decision-making in athlete care [7,10].

Across the core domains of sports medicine, AI functions primarily as a decision-support layer rather than an autonomous decision-maker [10]. By processing longitudinal physiological, biomechanical, imaging, and behavioral data, AI-based systems generate probabilistic outputs that may inform risk stratification, diagnostic interpretation, recovery monitoring, and RTP assessment. However, the clinical relevance of these systems depends less on algorithmic performance alone than on how outputs are interpreted and integrated into real-world decision-making processes [11].

Importantly, AI does not eliminate clinical uncertainty; rather, it redistributes it. Algorithmic predictions are inherently probabilistic, context-dependent, and limited by data quality, model design, and external validity. High predictive accuracy does not necessarily translate into improved clinical outcomes, particularly in complex and high-stakes decisions such as RTP clearance. Misinterpretation of predictive scores, overreliance on automated outputs, and limited generalizability across athletic populations represent key risks associated with AI integration [4]. In this evolving paradigm, the role of the sports physician shifts toward that of a clinical integrator. The physician is responsible for contextualizing AI-derived outputs within athlete-specific characteristics, sport-specific demands, and broader ethical considerations that cannot be fully captured by computational models alone [12]. This integrative function is essential in decision contexts where uncertainty, risk tolerance, and long-term athlete welfare must be carefully balanced.

To structure this transformation, the following sections examine key domains in which AI is increasingly integrated into sports medicine practice. For each domain, four core questions are addressed:

1. Which clinical decision is being augmented?
2. What does AI add compared with traditional practice?
3. Which limitations or risks emerge from AI integration?
4. Why does physician oversight remain essential?

**Figure 1:**
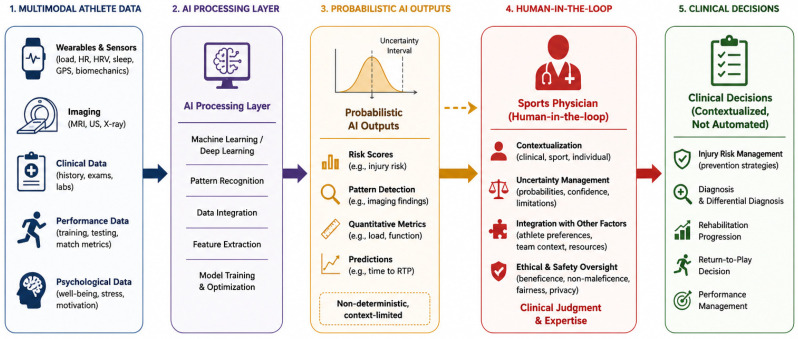
Decision-centered AI framework in sports medicine. Artificial intelligence (AI) processes multimodal athlete data into probabilistic outputs that support—but do not determine—clinical decisions. These outputs remain non-deterministic and context-limited, requiring interpretation by the sports physician acting as a human-in-the-loop integrator. The physician contextualizes AI-derived information within clinical, sport-specific, and ethical frameworks, ensuring that final decisions are individualized and not algorithm-driven.

Using this framework, the subsequent sections examine key areas in which AI is being integrated into sports medicine practice, including injury risk stratification and prevention, musculoskeletal imaging, rehabilitation monitoring, RTP decision-making, and clinical workflow support. Figure 1 summarizes this conceptual model, illustrating how AI integrates multimodal data into probabilistic outputs that support—but do not determine—clinical decisions, with the sports physician acting as a human-in-the-loop integrator.

### 3.1. Risk Stratification and Injury Prevention

Injury prevention represents one of the most clinically consequential applications of AI in sports medicine, as it directly targets a core medical decision: the longitudinal stratification of injury risk and the implementation of preventive interventions. Traditional prevention strategies have relied on retrospective analyses, periodic screening, and subjective clinical judgment, often identifying elevated risk only after maladaptive load–response patterns have already developed [11,13,14]. In contrast, AI enables continuous, individualized risk profiling by integrating dynamic, heterogeneous data streams, thereby supporting earlier, more adaptive preventive strategies. From a clinical perspective, the primary contribution of AI lies not in deterministic injury prediction, but in modeling complex, non-linear interactions among training load, physiological response, and tissue tolerance. AI-based systems can integrate external load metrics (e.g., distance covered, accelerations, jumps), internal load indicators (e.g., heart rate–derived measures and perceived exertion), recovery parameters (e.g., sleep quality), psychological stress, and prior injury history to generate probabilistic estimates of injury susceptibility rather than relying on static thresholds [7,11,15,16,17,18,19,20]. Within this framework, variables such as the acute–chronic workload ratio, neuromuscular asymmetries, and sleep disturbances should be interpreted as dynamic contributors to risk rather than fixed clinical cut-offs.

Closely related to injury prevention is load management, where the clinical objective is to balance training stimulus and recovery while minimizing injury risk. AI-based systems extend beyond descriptive monitoring by identifying deviations from an athlete’s individual baseline that may signal maladaptation, excessive fatigue, or increased vulnerability to injury [15,21,22,23,24,25,26,27,28]. By integrating longitudinal physiological, biomechanical, and psychophysiological data—including heart rate variability, perceived fatigue, mood, and sleep quality—these approaches support a more individualized interpretation of training response. In this context, performance optimization and injury prevention represent interconnected components of the same continuum, in which training decisions are guided by data-informed risk estimation [29,30,31].

Empirical evidence suggests that ML models can achieve moderate-to-high discriminatory performance in injury prediction, although results vary substantially depending on sport, dataset size, and outcome definitions [6,7,16,32,33,34,35,36,37]. Reported area-under-the-curve (AUC) values typically range from approximately 0.70 to 0.85, with ensemble methods often outperforming traditional statistical approaches in selected contexts [7,16,35]. For instance, large-scale analyses in professional baseball and elite athlete cohorts have demonstrated improved predictive performance compared with conventional regression models, particularly when multimodal data are incorporated [36]. However, these findings remain highly context-dependent and are frequently derived from retrospective datasets.

Importantly, predictive performance does not necessarily translate into clinical utility. Injury prediction models are commonly affected by class imbalance, reflecting the relatively low incidence of injuries compared with exposure time, which may lead to inflated accuracy but clinically relevant false-positive rates. Moreover, models developed within single teams or specific competitive environments often exhibit limited external validity when applied across different populations or sports [16,38]. As a result, probabilistic risk estimates must be interpreted cautiously and integrated within a broader clinical decision-making framework rather than used as standalone decision tools.

Several additional limitations constrain the translation of AI-based injury risk stratification into routine practice. Data heterogeneity across wearable platforms and monitoring systems reduces reproducibility, while inconsistencies in injury definitions and potential label leakage in retrospective datasets may further compromise model validity [16,38].

Model interpretability remains a critical challenge, particularly for DL architectures. Although explainable AI techniques such as SHAP and LIME can provide insights into feature importance, their clinical applicability remains limited and requires careful interpretation [22]. Ethical considerations also play a relevant role, including data ownership, informed consent, and the potential misuse of probabilistic risk scores in decisions affecting training exposure, team selection, or contractual outcomes [39].

Taken together, AI-based injury risk stratification should be viewed as a tool for augmenting—rather than replacing—clinical reasoning. Its primary value lies in supporting dynamic risk awareness and guiding individualized preventive strategies, while acknowledging that uncertainty, context, and clinical judgment remain central to safe and effective decision-making.

### 3.2. Diagnostic Augmentation in Musculoskeletal Imaging

Musculoskeletal imaging represents a central pillar of sports medicine, informing diagnosis, monitoring, and RTP decisions across a wide spectrum of acute and chronic conditions [13,40]. In this context, the most clinically relevant contribution of AI lies in diagnostic augmentation rather than replacement. AI-based systems primarily enhance the consistency, speed, and quantitative depth of image interpretation, particularly in high-performance environments where timely and reproducible assessments are essential [41,42].

From a decision-making perspective, the key question is not merely whether an abnormality is present, but how imaging findings are detected, quantified, and integrated into longitudinal athlete management. DL models—most commonly convolutional neural networks—have demonstrated strong performance in several musculoskeletal imaging tasks, including fracture detection, ligament injury classification, and automated anatomical segmentation across radiography, ultrasound, computed tomography (CT), and magnetic resonance imaging (MRI) [43,44,45]. In selected applications, AI-assisted interpretation has been shown to improve diagnostic sensitivity and reduce reading time, particularly in high-throughput settings such as emergency radiology [46,47]. For example, AI-based tools for wrist and hand radiographs have demonstrated improved sensitivity for subtle or occult fractures compared with standard interpretation alone, supporting a role as second-reader systems that may reduce diagnostic oversight [48].

**Figure 2:**
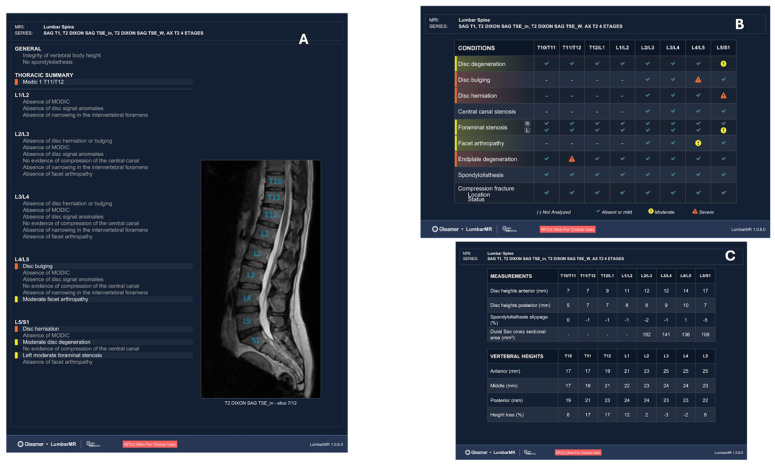
AI-augmented musculoskeletal imaging workflow in sports medicine. Artificial intelligence supports musculoskeletal imaging by enabling automated detection, structured reporting, and quantitative analysis of imaging data. Panel (**A**) illustrates AI-generated structured reporting of lumbar spine MRI findings across spinal levels. Panel (**B**) shows automated detection and grading of abnormalities. Panel (**C**) presents quantitative measurements derived from AI-based segmentation, including disk height, vertebral body dimensions, and dural sac cross-sectional area.

Beyond detection, AI is increasingly enabling automated quantitative analysis, transforming imaging from a descriptive modality into a semi-quantitative and longitudinal monitoring tool. Recent architectures enable automated measurement of parameters such as joint-space narrowing, spinal alignment, muscle volume, tendon thickness, and cartilage morphology on MRI and CT [44,45]. These capabilities are particularly relevant in sports medicine, where side-to-side comparisons and temporal changes often guide rehabilitation progression and RTP decisions. By reducing inter-observer variability and improving measurement reproducibility, AI-assisted imaging may support more standardized and objective assessment of structural recovery (Figure 2).

However, diagnostic accuracy alone does not define clinical utility in sports medicine [47]. Structural abnormalities frequently persist in asymptomatic athletes, and imaging findings do not necessarily correlate with symptoms, functional capacity, or readiness to return to sport. Current AI systems—largely trained on isolated imaging data—remain unable to integrate critical contextual variables such as injury mechanism, sport-specific load exposure, positional demands, symptom evolution, and rehabilitation stage, all of which are central to clinical decision-making [42,46]. Consequently, high model performance does not necessarily translate into improved clinical outcomes, particularly in complex, context-dependent decisions such as RTP clearance.

To provide a more structured overview of the available evidence, representative studies of AI-assisted musculoskeletal imaging are summarized in Table 1. As shown, reported performance is often encouraging for narrowly defined tasks such as fracture detection, knee lesion classification, or accelerated MRI reconstruction; however, methodological heterogeneity, limited external validation, and scarce athlete-specific datasets constrain direct comparisons and reduce certainty about clinical generalizability.

Several limitations further constrain the translation of AI-assisted musculoskeletal imaging into routine practice. Many algorithms are trained on curated datasets that do not reflect the variability of real-world imaging protocols, scanner types, or athletic populations, limiting external validity [42,49,50]. Rare or sport-specific injury patterns remain underrepresented, and model performance may degrade in out-of-distribution settings. Interpretability also remains a challenge, particularly for DL architecture. Although explainable AI techniques such as SHAP and LIME can provide insights into feature relevance, their clinical integration remains inconsistent and requires careful interpretation [22]. Additional barriers include workflow integration, cost considerations, and the need for clinician training in interpreting AI outputs.

As illustrated in Figure 2, AI-assisted imaging may support three complementary functions: automated detection of abnormalities, structured reporting, and quantitative assessment of imaging-derived parameters. However, these outputs remain intermediate analytic products rather than clinical decisions, and their interpretation requires integration with symptoms, functional findings, and sport-specific context. Taken together, AI in musculoskeletal imaging should be considered a decision-support technology that enhances diagnostic consistency and quantitative assessment, rather than a stand-alone determinant of diagnosis or RTP clearance. Its clinical value ultimately depends on integration within a broader, context-aware decision-making process led by the sports physician.

**Table 1:** Representative studies of AI applications in musculoskeletal imaging relevant to sports medicine.

Study	Clinical Task	Imaging Modality	AI Approach	Main Performance |Metric(s)/Main Finding	Validation Setting	Methodological Considerations/ Limitations	Level of Evidence (Interpretative) *	Relevance to Sports Medicine
Duron et al., 2021 [46]	Detection/localization of appendicular fractures	Radiography	Commercial DL-based fracture detection aid	AI assistance increased reader sensitivity by 8.7% and specificity by 4.1%, without loss of reading speed	Multicenter cross-sectional reader study	Acute trauma setting; not athlete-specific; evaluates reader augmentation rather than autonomous deployment	●●●	Relevant for same-day sport trauma triage, especially missed fractures
Guermazi et al., 2022 [47]	Fracture recognition across multiple skeletal regions	Radiography	DL-based detection support	AI improved fracture detection sensitivity across most regions and did not lengthen reading time; patient-level gain significant in most anatomical regions	Multireader validation study	Performance depends on body region and case mix; trauma workflow rather than longitudinal athlete follow-up	●●●	Strong relevance for sideline-to-imaging acute injury pathways
Jacques et al., 2024 [48]	Wrist/hand fracture detection using CT-based ground truth	Radiography	Commercial AI algorithm	AI improved radiologists’ sensitivity for wrist/hand fractures versus standard reading, with a CT reference standard	Comparative reader study	Focused anatomical task; may not generalize to other injury types or athlete-adapted bone morphology	●●○	High relevance in athletes with subtle carpal/metacarpal trauma
Liu et al., 2019 [51]	Complete ACL tear detection	Knee MRI	Deep CNN pipeline	Reported AUC ~0.98 with sensitivity ~0.96 and specificity ~0.96 in internal testing	Retrospective, internally validated dataset	Highly task-specific; likely optimized imaging protocol; limited evidence on external generalizability and partial tears	●○○	Relevant to sport knee injury diagnosis, but not sufficient alone for RTP decisions
Astuto et al., 2021 [52]	Detection and grading of cartilage, bone marrow, meniscal, and ACL abnormalities	Knee MRI	3D deep learning	High sensitivity/specificity/accuracy reported for lesion-severity scoring; DL assistance also improved interreader agreement	Retrospective study using 1435 MRI exams derived from prior datasets	Knee-focused; reader-assistance paradigm; limited sport-specific validation; broad lesion label quality may influence outputs	●●○	Relevant for the structured interpretation of complex post-traumatic knee MRI
Thomas et al., 2020 [53]	Automated staging of radiographic knee osteoarthritis severity	Radiography	Deep neural network	Performance comparable to fellowship-trained musculoskeletal radiologists for KL severity staging	Large retrospective dataset (Osteoarthritis Initiative)	Degenerative rather than athletic population; indirect sports relevance; classification task may not map cleanly to symptoms/function	●●○	More relevant to long-term joint health than acute athlete imaging
Hahn et al., 2022 [54]	Accelerated shoulder MRI with preserved diagnostic quality	Shoulder MRI	Deep learning–based image reconstruction (DLR)	67% scan-time reduction with similar subjective image quality, artifacts, and diagnostic performance compared with standard sequences	Comparative diagnostic study	Reconstruction study, not a lesion-detection model; focuses on efficiency and image quality rather than outcome prediction	●●●	Highly relevant for improving workflow in the shoulder imaging of athletes
Wu et al., 2025 [55]	Torn vs intact rotator cuff tendon detection	Ultrasound	YOLOv7-CBAM attentional DL model	Reported high diagnostic accuracy and improved interobserver reliability for ultrasound-based rotator cuff tear detection	Single-center diagnostic dataset with MRI comparison	Early evidence; operator dependence and acquisition variability remain major barriers; no clear external validation	●○○	Relevant because the US is widely used in team and outpatient sports medicine
Scott et al., 2024 [56]	Automated tendon segmentation to quantify structural change in tendinopathy	Ultrasound	Texture-based segmentation using GLCM + hidden Gaussian Markov random fields	Demonstrated feasibility of automated tendon segmentation for quantitative tendon assessment	Development/feasibility study	Quantification-focused, not full clinical diagnosis; external clinical validation lacking	●○○	Potentially useful for longitudinal monitoring of tendinopathy in athletes

* Level of evidence (interpretative): ●●● high (prospective and/or multicenter validation); ●●○ moderate (retrospective with validation or large datasets); ●○○ low (retrospective, feasibility, or narrative evidence).

### 3.3. Rehabilitation and Functional Recovery

Rehabilitation represents a critical phase in sports medicine, during which clinical decisions directly influence recovery quality, reinjury risk, and the timing of RTP [57,58,59]. Traditionally, rehabilitation strategies have relied on standardized protocols, periodic clinical reassessment, and relatively coarse functional milestones [60,61]. AI-enabled technologies are progressively shifting this paradigm toward data-driven, adaptive rehabilitation pathways, enabling continuous monitoring of functional recovery and more individualized progression based on objective performance metrics rather than fixed time-based criteria [62,63].

From a clinical perspective, the key decision being augmented is not the selection of individual therapeutic exercises, but the modulation of rehabilitation load, progression, and readiness in response to ongoing functional feedback. AI-assisted rehabilitation systems can integrate multimodal data streams—including wearable sensors, inertial measurement units, pressure sensors, and computer vision-based motion analysis—to quantify movement quality, symmetry, coordination, and load distribution during rehabilitation tasks [64]. These data can be combined with physiological and subjective indicators, such as heart rate variability, perceived exertion, pain, and fatigue, to generate a more granular and dynamic assessment of recovery status and tolerance to rehabilitation load [12,64,65].

A particularly relevant contribution of AI lies in the detection of subtle compensatory strategies and neuromuscular asymmetries that may escape conventional clinical observation [66]. Through longitudinal movement analysis, AI models can identify deviations from an athlete’s baseline motor patterns, enabling earlier corrective interventions. This is especially relevant following complex injuries, such as anterior cruciate ligament reconstruction, where persistent asymmetries in biomechanics and load distribution may remain despite apparent clinical recovery [67].

Emerging evidence supports the use of wearable-derived biomechanical data combined with ML approaches to estimate tissue loading and monitor functional recovery. For example, sensor-based models have been used to estimate tibial loading during running, providing a proxy for tissue stress that may inform load progression strategies [68]. In parallel, digital rehabilitation platforms incorporating remote monitoring and automated feedback have demonstrated comparable outcomes to conventional physiotherapy in selected randomized studies, while improving adherence and accessibility [69]. However, these results remain context-dependent and are often derived from controlled or non-elite populations.

Importantly, improvements in measurement precision or monitoring capability do not necessarily translate into improved clinical outcomes. While AI systems can enhance detection of biomechanical deficits and provide continuous feedback, there is limited prospective evidence demonstrating that AI-guided rehabilitation strategies reduce reinjury rates or accelerate safe RTP in athletic populations [20]. As a result, AI-derived outputs should be interpreted as decision-support signals rather than prescriptive recommendations.

In clinical practice, these technologies are most effective within hybrid rehabilitation models, where remote monitoring complements direct clinical supervision. AI-assisted telerehabilitation may improve adherence, accessibility, and longitudinal follow-up, but critical decisions—such as load progression, return to sport-specific training, and RTP clearance—require clinical judgment and contextual interpretation by the sports physician [70].

Several limitations continue to constrain widespread implementation. Many AI-driven rehabilitation models are developed using small or homogeneous cohorts, limiting generalizability across sports and performance levels [11,16]. Algorithmic outputs are often insufficiently transparent, potentially reducing clinician and athlete trust [4]. Interoperability between wearable devices, rehabilitation platforms, and electronic health records remains limited, and financial or infrastructural barriers may restrict access outside elite environments [71]. These challenges highlight the need for robust, prospective validation studies assessing not only model performance but also clinical impact [20]. Taken together, AI in rehabilitation should be viewed as a tool for enhancing the resolution and continuity of functional assessment, rather than as a replacement for clinical decision-making. Its primary value lies in supporting adaptive, individualized rehabilitation pathways, while acknowledging that uncertainty, context, and clinician expertise remain central to safe recovery and RTP decision-making.

### 3.4. Return-to-Play Decision-Making

Return-to-play (RTP) decisions are among the most complex and high-stakes responsibilities in sports medicine, requiring the integration of medical recovery, functional readiness, psychological preparedness, and sport-specific demands [72]. Unlike diagnostic or rehabilitative decisions, RTP clearance carries immediate implications for athlete safety, performance, and long-term career trajectory. In this context, AI functions as a decision-support tool that provides objective and longitudinal insights but does not replace clinical judgment or professional accountability [5].

From a decision-making perspective, the key question is not whether an athlete can return to sport, but how readiness is defined, quantified, and interpreted under conditions of uncertainty. Traditional RTP frameworks have relied on time-based criteria or isolated functional tests, which may inadequately capture the dynamic and multidimensional nature of recovery [73]. AI-based approaches instead leverage longitudinal and multimodal data to support individualized readiness assessment based on recovery trajectories rather than predefined timelines. Because validated AI models specifically designed for RTP clearance remain scarce, Table 2 includes representative AI and data-driven studies that inform RTP-related risk stratification, functional assessment, or decision-support frameworks.

Multimodal RTP systems can integrate data from force plates, wearable sensors, motion capture technologies, and validated psychological instruments to construct comprehensive readiness profiles [74]. Domains commonly assessed include strength and power asymmetries, jump and landing mechanics, joint range of motion, proprioceptive control, movement variability, and athlete-reported confidence or fear of reinjury [11,74,75,76]. These integrated datasets enable the detection of residual functional deficits that may not be evident through isolated clinical testing.

Computer vision approaches further expand the feasibility of movement analysis by enabling markerless motion capture from standard video recordings, including smartphone-based acquisition [77,78]. Automated video analysis has demonstrated the ability to identify movement asymmetries and biomechanical patterns associated with reinjury risk or incomplete recovery, potentially extending biomechanical assessment beyond specialized laboratory settings [40]. AI-based RTP models have been explored across multiple injury contexts. In anterior cruciate ligament reconstruction, ML models integrating biomechanical and functional data have shown potential for identifying persistent asymmetries and modeling recovery trajectories [36,79]. Similar approaches have been applied to hamstring injuries, ankle sprains, and sport-related concussion, incorporating fatigue metrics, joint loading patterns, and sport-specific movement demands to better approximate real-world exposure [80]. Emerging systems are also beginning to incorporate contextual variables such as sport discipline, playing position, and exposure risk, reinforcing the notion that RTP is not a fixed endpoint but a context-dependent clinical decision [81].

However, model performance does not equate to decision validity. RTP remains inherently probabilistic, and AI-derived outputs are constrained by heterogeneous inputs, inconsistent outcome definitions, and limited external validation [11,50]. Predictive models may generate apparently precise readiness scores while failing to capture critical contextual factors such as psychological readiness, environmental stressors, team dynamics, or competitive pressure [10]. This creates a potential mismatch between algorithmic outputs and real-world decision requirements.

From a clinical standpoint, a key risk is overinterpreting quantitative thresholds as definitive indicators of readiness. AI systems may improve measurement precision and data integration, but they do not define acceptable risk. RTP decisions inherently involve trade-offs between performance, reinjury risk, and long-term athlete health, and these trade-offs cannot be fully encoded within algorithmic models. Consequently, reliance on AI-derived scores without appropriate contextualization may lead to premature or inappropriate return decisions.

A conceptual framework for AI-assisted RTP decision-making can therefore be described as a three-step process: (1) data integration and probabilistic risk estimation, (2) contextual interpretation incorporating athlete-specific and sport-specific factors, and (3) shared clinical decision-making under uncertainty. Within this framework, AI contributes primarily to the first step, while the latter stages remain fundamentally clinician-driven.

For these reasons, AI should be considered a tool to structure and inform RTP decisions rather than to automate clearance. The sports physician remains responsible for integrating objective data with clinical examination, athlete-reported outcomes, and contextual variables, ensuring that RTP decisions are individualized, ethically sound, and aligned with both short- and long-term athlete welfare [11].

**Table 2:** Representative AI and data-driven studies informing return-to-play (RTP) decision-making in sports medicine.

Study	Clinical Context	Data Sources	AI/Data-Driven Approach	Main Performance Metric(s)/Main Finding	Validation Setting	Level of Evidence (Interpretative) *	Limitations	Relevance to RTP
Jauhiainen et al., 2022 [36]	ACL injury risk (elite female athletes)	Screening test battery (strength, biomechanics)	ML (multiple models)	AUC values up to ~0.79 for ACL injury prediction, depending on model configuration; moderate discrimination in identifying athletes at increased injury risk.	Prospective cohort	●●●	Focus on injury prediction, not RTP; no direct readiness outcome	Indirect relevance: informs RTP risk stratification
Karnuta et al., 2020 [35]	Injury prediction (MLB players)	Performance + injury history	ML vs regression	Machine learning models outperformed traditional regression approaches in predicting next-season injury risk, with improved predictive accuracy across large-scale retrospective data.	Large retrospective dataset	●●○	Not RTP-specific; population = baseball players	Relevant for post-RTP risk estimation
Desai, 2024 [10]	AI in RTP decision-making	Multimodal (wearables, biomechanics, context)	Narrative synthesis of AI approaches	No original performance metrics; narrative synthesis highlighting the potential of AI to support RTP decision-making through multimodal data integration.	Narrative review	●○○	No performance metrics; heterogeneous evidence	Framework-level relevance
Leckey et al., 2024 [15]	Injury risk modeling	Load, physiological, and contextual variables	ML (review)	No pooled performance metric; included studies showed highly variable model performance, with limited external validation and inconsistent methodological quality.	Systematic/scoping review	●○○	Not RTP-specific; heterogeneity of methods	Supports probabilistic risk framing in RTP decisions

* Level of evidence (interpretative): ●●● high (prospective and/or multicenter validation); ●●○ moderate (retrospective with validation or large datasets); ●○○ low (retrospective, feasibility, or narrative evidence). AI, artificial intelligence; ML, machine learning; ACL, anterior cruciate ligament; RTP, return to play; MLB, Major League Baseball.

Beyond diagnostic and therapeutic decisions, AI is increasingly influencing the organizational and cognitive dimensions of sports medicine practice. In clinical environments characterized by growing administrative burden and expanding multimodal data streams—from imaging and wearable monitoring to performance metrics and electronic health records—AI-driven tools are being developed to support workflow efficiency, information synthesis, and decision consistency. The primary objective of these technologies is not to replace physician judgment, but to preserve clinician time and cognitive resources for direct athlete care [5].

From a clinical perspective, the decision being augmented is not medical reasoning itself, but the prioritization, synthesis, and presentation of clinically relevant information in time-sensitive, data-dense environments [6,7]. AI systems applied to workflow optimization typically rely on natural language processing for documentation automation, ML-based clinical decision support systems (CDSS) for pattern recognition and safety alerts, and multimodal data integration to aggregate heterogeneous inputs—including imaging, wearable data, laboratory findings, and athlete-reported outcomes—into structured clinical summaries [3,82,83,84,85].

Large language models (LLMs) are emerging as supportive tools capable of summarizing longitudinal clinical records, facilitating rapid evidence retrieval, and assisting with report drafting or educational tasks in high-volume clinical settings [77]. In sports medicine, these tools may support tasks such as synthesizing athletes’ medical histories, generating structured reports, or assisting clinicians in navigating complex multidisciplinary datasets. While these applications may improve efficiency and accessibility of information, LLM outputs remain probabilistic and may be affected by hallucinations, incomplete contextual understanding, and variable reliability depending on input quality [74,86]. As such, their role should be considered assistive rather than authoritative.

In practical settings, AI-enabled workflow systems may improve standardization and longitudinal tracking of athlete health data. CDSS platforms can assist clinicians by identifying deviations from expected recovery trajectories, flagging potential safety concerns, and supporting structured second-opinion processes during time-constrained evaluations [87]. At the team level, integrated dashboards combining clinical, rehabilitation, and performance data may enhance multidisciplinary communication among physicians, physiotherapists, strength coaches, and sport scientists, facilitating coordinated and data-informed athlete management strategies [88,89].

However, several limitations constrain the effective implementation of AI-driven workflow solutions. Model performance is highly dependent on data quality, and incomplete or inconsistent inputs may generate misleading or clinically inappropriate outputs [6,90,91]. Overreliance on automated recommendations introduces the risk of automation bias, particularly when uncertainty and model limitations are not explicitly communicated. LLM-based systems may struggle with sport-specific terminology, contextual nuance, and multilingual clinical environments, potentially affecting reliability in real-world settings. Additional barriers include integration within existing electronic health record infrastructures, financial costs, and regulatory challenges related to data governance and algorithm accountability [6,90,91]. Importantly, improved efficiency or information accessibility does not necessarily translate into improved clinical decision-making. While AI systems may enhance data organization and reduce cognitive load, they may also introduce new failure modes, including oversimplification of information, loss of contextual nuance, and misplaced trust in automated outputs. Consequently, AI-enabled workflow tools should be viewed as cognitive support systems that augment—but do not replace—clinical reasoning.

**Figure 3:**
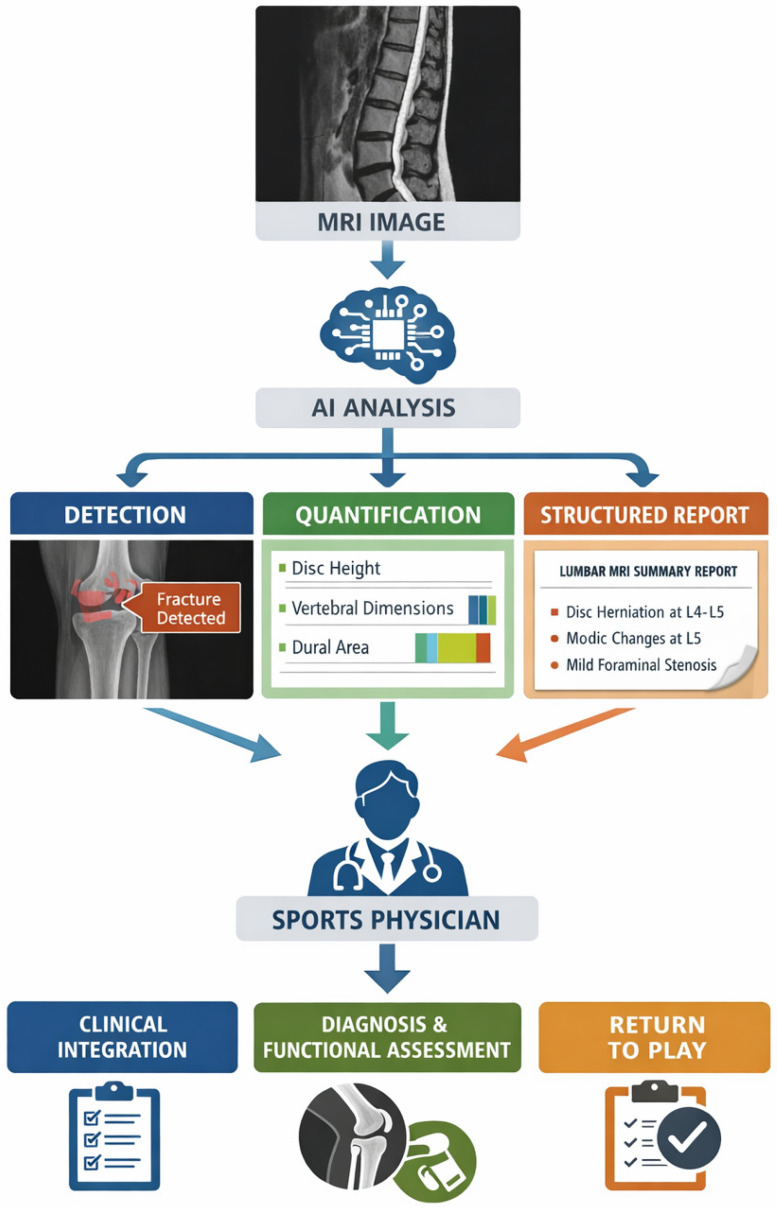
The sports physician within an AI-augmented sports medicine ecosystem. AI systems generate structured outputs from multimodal clinical and performance data, while the sports physician remains responsible for their validation, contextualization, and translation into clinical decisions.

Figure 3 illustrates this workflow-oriented perspective, in which AI contributes to data aggregation, structured outputs, and cognitive support, but does not independently generate clinical decisions. Potential failure modes—including automation bias, hallucinations in language models, and loss of contextual nuance—must therefore be recognized and actively mitigated by the physician. Within this framework, the sports physician remains the central decision-maker, responsible for validating, contextualizing, and integrating AI-generated outputs into individualized and clinically appropriate decisions. Effective use of these tools requires not only technical integration but also the development of new competencies related to critical appraisal of AI outputs, uncertainty management, and interdisciplinary collaboration within AI-augmented clinical environments.

## 4. Artificial Intelligence-Enabled Sports Medicine Devices

Although the number of AI-enabled medical devices approved for clinical use has increased substantially over the past decade, only a limited proportion of these technologies are directly tailored to sports medicine practice. Most currently authorized systems have been developed within radiology, primarily focusing on automated image interpretation, detection of structural abnormalities, and quantitative measurements. Additional emerging applications include wearable-based biomechanical monitoring systems, balance assessment platforms, and AI-assisted imaging reconstruction technologies [92,93].

From a regulatory perspective, the majority of these devices have obtained clearance through the U.S. Food and Drug Administration (FDA) 510(k) pathway, indicating substantial equivalence to existing technologies rather than demonstration of superior clinical performance. Only a minority have been authorized through the De Novo pathway, which reflects greater novelty but does not necessarily imply robust clinical validation in athlete-specific populations. Consequently, regulatory approval should not be interpreted as evidence of clinical effectiveness in sports medicine contexts.

Table 3 provides selected examples of AI-enabled medical devices with potential relevance to sports medicine, highlighting their clinical domain, primary function, and regulatory status. This overview is not intended to be exhaustive, but rather to illustrate the current technological landscape and its translational potential.

A critical observation is that most available systems address isolated components of the clinical pathway—such as fracture detection, image reconstruction, or biomechanical assessment—rather than providing integrated, athlete-centered decision support. While these tools may improve diagnostic efficiency, standardization, and measurement reproducibility, their impact on clinically meaningful outcomes—such as return-to-play decisions, injury prevention, or long-term athlete health—remains largely unproven.

Furthermore, the majority of AI-enabled devices have been developed and validated using general population datasets, with limited representation of athletic populations. This raises concerns about external validity, particularly regarding sport-specific physiological adaptations, injury patterns, and performance demands. For example, musculoskeletal imaging algorithms may not adequately differentiate between physiological adaptations (e.g., tendon thickening, cortical remodeling) and pathological findings in athletes, potentially leading to overdiagnosis or misinterpretation.

Another key limitation is the lack of longitudinal validation. Many devices provide cross-sectional outputs that do not account for temporal dynamics, which are central to decision-making in sports medicine. In particular, their integration into rehabilitation monitoring and return-to-play frameworks remains fragmented and insufficiently standardized.

Despite these limitations, AI-enabled devices represent a significant step toward quantifying and objectifying clinical data in sports medicine. When appropriately integrated into clinical workflows, these tools may enhance measurement consistency, reduce diagnostic variability, and support data-driven decision-making. However, their clinical adoption should be guided by a critical appraisal of their validation frameworks, intended use, and applicability to athlete-specific populations.

- Prospective clinical study = highest level in this context;
- Multicenter validation = moderate;
- Retrospective validation = limited;
- Limited/unclear validation = weak evidence.

## 5. Ethical, Professional, and Organizational Implications of AI in Sports Medicine

The integration of AI into sports medicine represents not only a technological advancement but a structural transformation with ethical, professional, and organizational implications [94]. As AI systems increasingly inform diagnostic workflows, rehabilitation monitoring, injury risk estimation, and RTP decision-making, they extend into domains traditionally governed by clinical expertise, professional accountability, and contextual judgment [1,2].

A central challenge relates to the interpretability, reliability, and uncertainty of AI-generated outputs [2]. Many contemporary models operate as partially opaque systems, providing probabilistic outputs without transparent reasoning. In high-stakes sports medicine decisions—such as RTP clearance—this lack of interpretability complicates informed consent and shared decision-making. The primary clinical risk is not solely algorithmic error, but inappropriate trust in outputs whose uncertainty, limitations, and context-dependence are insufficiently understood or communicated. This is particularly relevant for LLMs, which may generate plausible but factually incorrect or contextually inappropriate information, reinforcing the need for critical appraisal of AI-generated content.

Data governance represents a further critical dimension [95]. Modern sports medicine increasingly relies on continuous, granular data from wearable devices, imaging systems, performance-tracking platforms, and digital health applications. These data streams raise complex questions regarding data ownership, access rights, secondary use, commercialization, and long-term storage. In elite sport environments, these issues extend beyond healthcare into performance optimization and contractual decision-making. Sports physicians are therefore not only clinicians but also data stewards, responsible for ensuring privacy protection, proportional data use, and preservation of athlete autonomy in contexts where AI-derived insights may influence training availability, team selection, or career trajectories [95].

Bias and equity represent additional challenges [96]. Many AI systems are developed using datasets that disproportionately represent male, elite, and Western athletic populations. Without deliberate efforts to ensure diversity in training data and to continuously monitor for bias, AI tools risk reinforcing existing disparities in sports medicine care. This may lead to reduced accuracy or inappropriate recommendations in underrepresented groups, including female athletes, youth athletes, para-athletes, and individuals competing outside professional sport environments [1]. Importantly, these biases may not be immediately apparent, further emphasizing the need for systematic validation across diverse populations.

Regulatory frameworks remain in evolution and are not yet fully aligned with the specific needs of sports medicine [97]. Although an increasing number of AI-enabled medical devices have obtained regulatory clearance—primarily through the U.S. Food and Drug Administration (FDA) 510(k) pathway—such approval typically reflects technical equivalence rather than demonstrated clinical effectiveness or impact on athlete outcomes. Current regulatory models are also challenged by adaptive algorithms and continuous-learning systems, which may evolve after deployment. Moreover, existing frameworks rarely address downstream clinical consequences of AI-assisted decision-making, including medico-legal responsibility in cases of adverse outcomes [97].

Beyond technical performance, the integration of AI has important implications for professional roles and responsibilities in sports medicine. As AI systems increasingly inform clinical workflows and decision-making processes, the role of the sports physician evolves toward that of a critical integrator of algorithmic outputs. This shift requires competencies that extend beyond traditional clinical expertise.

At an organizational level, implementing AI requires structural adaptation, including integration with electronic health record systems, interdisciplinary collaboration, and investment in digital infrastructure. These challenges are particularly relevant outside elite professional environments, where access to advanced technologies may be limited, potentially widening disparities in care. An additional and often overlooked dimension relates to the economic sustainability of AI integration in sports medicine. Despite increasing technical performance and regulatory approval of AI-enabled tools, robust cost-effectiveness analyses remain scarce, particularly in athlete-specific populations. Most available studies focus on diagnostic accuracy or workflow efficiency, without evaluating downstream clinical outcomes, resource utilization, or long-term impact on athlete health. As a result, the economic value of AI-assisted decision-making in sports medicine remains uncertain, and its large-scale implementation should be approached with caution until supported by prospective health economic evaluations.

Ultimately, the responsible integration of AI in sports medicine depends on maintaining a clear ethical hierarchy in which athlete welfare remains the primary objective. AI systems should be implemented as tools to support—not replace—clinical judgment, within transparent frameworks of validation, accountability, and oversight. Ensuring this balance will require coordinated efforts from clinicians, researchers, regulatory bodies, and sporting organizations to develop standards that are not only technologically robust but also ethically and clinically grounded [9,98,99].

## 6. The Future Sports Physician: Skills and Roles

In AI-augmented sports medicine, the relevant question is no longer whether physicians remain central, but which competencies are required to use algorithmic outputs safely and effectively. These competencies include critical appraisal of AI systems, integration of multimodal data, management of uncertainty, contextual reasoning, and ethical communication [4,5].

In this context, the central challenge is not to redefine the role of the sports physician in abstract terms, but to operationalize the competencies required to function safely and effectively within AI-augmented clinical environments. These competencies extend beyond traditional clinical expertise and include the ability to critically appraise algorithmic outputs, integrate heterogeneous data sources, manage uncertainty, and ensure ethical and context-aware decision-making.

Importantly, AI does not reduce clinical complexity—it redistributes it. While algorithmic systems may improve data processing and pattern recognition, they also introduce new layers of abstraction and potential failure modes. As a result, the physician’s role increasingly centers on managing the interface between data, algorithms, and real-world decision-making, particularly in high-stakes contexts such as RTP clearance, where medical, performance, and career considerations intersect.

Table 4 summarizes these competency domains, linking each to practical responsibilities and potential risks if misapplied.

## 7. Conclusions

Artificial intelligence is progressively transforming sports medicine by reshaping how clinical data are generated, interpreted, and integrated into decision-making processes. Across injury prevention, musculoskeletal imaging, rehabilitation monitoring, RTP assessment, and clinical workflow management, AI functions primarily as a decision-support layer, enabling more granular, longitudinal, and data-driven evaluation of athlete health and performance.

However, integrating AI into clinical practice introduces new complexities rather than eliminating them. Algorithmic outputs remain probabilistic, context-dependent, and inherently limited by data quality, model design, and external validity. Importantly, improvements in model performance do not necessarily translate into improved clinical outcomes, particularly in high-stakes decisions such as RTP clearance.

In this context, the sports physician remains central as a human-in-the-loop decision-maker, responsible for critically interpreting AI-generated outputs, contextualizing them within the broader clinical and sport-specific environment, and managing uncertainty in a safe and ethically sound manner. The effective use of AI, therefore, depends not only on technological capability but also on the clinician’s ability to integrate data, recognize limitations, and balance competing risks and priorities.

Ultimately, the future of AI in sports medicine will be determined not by the sophistication of algorithms alone, but by the development of robust validation frameworks, the alignment of AI systems with real-world clinical decision-making, and the preservation of athlete-centered care as the guiding principle. In this evolving landscape, AI should be understood not as a replacement for clinical expertise, but as a tool that amplifies both the capabilities—and the responsibilities—of the sports physician.

## Figures and Tables

**Table 3:** Selected AI-enabled medical devices with potential relevance to sports medicine.

Device/Platform	Clinical Domain	Primary Function	AI Technology	Regulatory Status	Relevance to Sports Medicine	Validation Level *
**BoneView^®^ (Gleamer)**	MSK Radiography	Automated fracture detection on X-ray	CNN-based deep learning	FDA-cleared (510 [k])	Reduces missed fractures and supports rapid diagnosis in acute trauma	Retrospective + external validation
**TechCare Trauma (Milvue)**	Trauma radiology	Detection and localization of fractures and elbow effusion on X-ray	Deep learning	FDA 510(k) cleared	Facilitates rapid triage of skeletal injuries across multiple anatomical regions	Retrospective validation
**AZtrauma (AZmed/Nexus-MD)**	Trauma Radiology	Automated detection of skeletal abnormalities	Deep learning	FDA-cleared (510 [k])	Supports prioritization of imaging findings in acute injury settings	Limited published validation
**OsteoDetect**	MSK Radiography	Detection of distal radius fractures on wrist X-ray	Deep learning	FDA De Novo authorized	Supports the detection of subtle wrist fractures frequently seen in athletes	Prospective clinical study
**Aidoc C-Spine**	Spine CT	Automated cervical fracture detection	Deep learning	FDA 510(k) cleared	Assists in the rapid identification of cervical spine injuries in trauma	Multicenter validation
**Avicenna.AI**	Spine CT	Vertebral fracture detection	Deep learning	FDA 510(k) cleared	Improves detection of occult vertebral injuries	Retrospective + external validation
**Clarius MSK AI**	MSK Ultrasound	Automated identification and quantitative measurement of tendons and joints	ML-based segmentation	FDA-cleared (510 [k])	Enhances reproducibility of MSK ultrasound examinations	Limited clinical validation
**MuscleView™ 2.0**	MSK MRI	Automated muscle composition analysis	DL-based segmentation	FDA-cleared (510 [k])	Enables quantitative monitoring of muscle asymmetry during rehabilitation	Limited published validation
**SubtleMR**	MRI Workflow	Image denoising and accelerated MRI reconstruction	Deep Learning	FDA 510(k)	Enhances efficiency of MRI acquisition in clinical workflows	Multicenter validation
**ImageBiopsy Lab—LAMA/HIPPO/FROG**	MSK Imaging	Automated skeletal measurements (spine, hip, lower limb)	DL-based measurement algorithms	FDA-cleared (510 [k])	Supports objective longitudinal assessment of musculoskeletal structures	Limited validation
**AIR Recon DL (GE HealthCare)**	MRI workflow	AI-assisted MRI reconstruction and denoising	Deep learning reconstruction	FDA 510(k) cleared	Improves MRI acquisition speed and image quality	Multicenter validation
**Notch Motion System**	Motion Capture	Wearable IMU-based movement analysis	Sensor fusion + ML	FDA De Novo authorized	Provides objective movement metrics for rehabilitation and RTP monitoring	Early clinical validation
**Zibrio SmartScale**	Balance Assessment	Postural stability and fall-risk estimation	ML pattern recognition	FDA 510(k) cleared	Supports neuromotor and balance assessment	Limited validation
**PeekMed Web**	Orthopedic planning	AI-assisted surgical planning from imaging	AI-based image analysis	FDA 510(k) cleared	Supports orthopedic decision-making for hip, knee, and limb procedures	Limited clinical validation

AI, artificial intelligence; DL, deep learning; ML, machine learning; CNN, convolutional neural network; MSK, musculoskeletal; MRI, magnetic resonance imaging; CT, computed tomography; RTP, return to play; IMU, inertial measurement unit. * Validation Level (interpretative classification).

**Table 4:** Core competencies of the sports physician in AI-augmented sports medicine.

Domain	What AI Enables	Key Physician Responsibility	Clinical Risk If Misapplied
**AI literacy and critical appraisal**	Access to predictive models, performance metrics (e.g., discrimination, calibration), and automated outputs	Critically evaluate model validity, recognize bias, interpret uncertainty, and avoid inappropriate reliance on algorithmic outputs	Misinterpretation of outputs, overreliance on AI (automation bias), and inappropriate clinical decisions
**Integration of multimodal data**	Aggregation of imaging, wearable data, biomechanical metrics, rehabilitation parameters, and athlete-reported outcomes	Synthesize heterogeneous data into coherent clinical interpretation and individualized management strategies	Fragmented decision-making, overemphasis on isolated metrics, loss of clinical coherence
**Contextual clinical reasoning**	Structured and quantitative insights into physiological and functional parameters	Integrate sport-specific and contextual factors (e.g., competition demands, psychological readiness, environmental stressors, career implications) not captured by AI systems	Decontextualized decisions, inappropriate RTP clearance, neglect of psychosocial factors
**Ethical oversight and communication**	Risk stratification outputs, probabilistic predictions, and data-driven recommendations	Ensure transparency, protect data privacy, communicate uncertainty, and support shared decision-making with athletes and stakeholders	Loss of athlete trust, misuse of data, and ethically inappropriate decisions in high-stakes contexts
**Human–AI interaction and decision accountability**	Continuous AI support across diagnostic and monitoring workflows	Validate and take responsibility for final decisions, integrating AI outputs with clinical judgment and real-world context	Diffusion of responsibility, medico-legal vulnerability, and uncritical acceptance of AI recommendations

AI: artificial intelligence.

## Data Availability

Data are available on reasonable request to the corresponding author.
